# IGG4-SPECIFIC RESPONSES IN PATIENTS WITH *STAPHYLOCOCCUS AUREUS* BONE INFECTIONS ARE NOT PREDICTIVE OF POSTOPERATIVE COMPLICATIONS

**DOI:** 10.22203/eCM.v042a12

**Published:** 2021-09-22

**Authors:** J.R. Owen, M.P. Campbell, M.D. Mott, C.A. Beck, C. Xie, G. Muthukrishnan, J.L. Daiss, E.M. Schwarz, S.L. Kates

**Affiliations:** 1 Department of Orthopaedic Surgery, Virginia Commonwealth University, Richmond, VA, USA; 2 Center for Musculoskeletal Research, University of Rochester Medical Center, Rochester, NY, USA; 3 Department of Biostatistics and Computational Biology, University of Rochester Medical Center, Rochester, NY, USA

**Keywords:** Orthopaedic infections, immunoassay, *Staphylococcus aureus*, osteomyelitis, IgG4

## Abstract

The most prevalent pathogen in bone infections is *Staphylococcus aureus*; its incidence and severity are partially determined by host factors. Prior studies showed that anti-glucosaminidase (Gmd) antibodies are protective in animals, and 93.3 % of patients with culture-confirmed *S. aureus* osteomyelitis do not have anti-Gmd levels > 10 ng/mL in serum. Infection in patients with high anti-Gmd remains unexplained. Are anti-Gmd antibodies in osteomyelitis patients of the non-opsonising, non-complement-fixing IgG4 isotype? The relative amounts of IgG4 and total IgG against Gmd and 7 other *S. aureus* antigens: iron-surface determinants (Isd) IsdA, IsdB, and IsdH, amidase (Amd), *α*-haemolysin (Hla), chemotaxis inhibitory protein from *S. aureus* (CHIPS), and staphylococcal-complement inhibitor (SCIN) were determined in sera from healthy controls (Ctrl, *n* = 92), osteomyelitis patients whose surgical treatment resulted in infection control (IC, *n* = 95) or an adverse outcome (AD, *n* = 40), and post-mortem (PM, *n* = 7) blood samples from *S. aureus* septic-death patients. Anti-Gmd IgG4 levels were generally lower in infected patients compared to controls; however, levels among the infected were higher in AD than IC patients. Anti-IsdA, IsdB and IsdH IgG4 levels were increased in infected patients *versus* controls, and Jonckheere-Terpstra tests of levels revealed an increasing order of infection (Ctrl < IC < AD < PM) for anti-Isd IgG4 antibodies and a decreasing order of infection (Ctrl > IC > AD > PM) for anti-autolysin (Atl) IgG4 antibodies. Collectively, this does not support an immunosuppressive role of IgG4 in *S. aureus* osteomyelitis but is consistent with a paradigm of high anti-Isd and low anti-Atl responses in these patients.

## Editor’s note

The Scientific Editor responsible for this paper was Fintan Moriarty.

## Introduction

The need for novel approaches to address osteomyelitis remains a high priority, as it continues to be a major threat to successful outcomes of orthopaedic surgeries (Schwarz *et al*., 2019). *S. aureus* is the primary pathogen causing the greatest number of prosthetic-joint and fracture-related infections (Depypere *et al*., 2020; Goodson *et al*., 2020; Govaert *et al*., 2020; Kates and Tornetta, 2020). Treatment of this pathogen is particularly challenging given its ability to develop resistance to antibiotics (Assis *et al*., 2017; Kaplan, 2014), and the limitations of antibiotic-loaded bone cement are well-known (Schwarz *et al*., 2021). Thus, there is an urgent need for developing non-antibiotic, immunotherapeutic solutions to control this pathogen in bone infections.

Unfortunately, 17 anti-*S. aureus* vaccines have failed to demonstrate efficacy in clinical trials (Fowler and Proctor, 2014; Miller *et al*., 2020; Proctor, 2019). The most notable of them is the Merck vaccine based on iron-regulated surface determinant B (IsdB-V710). This vaccine conferred no protection, but heightened the risk of poor outcomes, including death, in patients who developed post-immunisation *S. aureus* infections (Fowler *et al*., 2013). It was surmised that non-neutralising pathogenic IsdB antibodies generated due to vaccination promoted *S. aureus* growth and dissemination into distal organs. Indeed, in a rodent *S. aureus* infection model, IsdB immunisations rendered mice more susceptible to multiple organ sepsis (Nishitani *et al*., 2020). Additionally, clinical studies revealed that patients who died from *S. aureus* osteomyelitis had the greatest elevation of anti-IsdB IgG levels (Nishitani *et al*., 2015). In contrast, the potential benefit of protective anti-*S. aureus* antibodies have also been demonstrated. For instance, anti-Gmd immunisation is a viable option for prevention and treatment of *S. aureus* osteomyelitis (Lee *et al*., 2020; Varrone *et al*., 2014; Varrone *et al*., 2011; Yokogawa *et al*., 2018). In an international retrospective study [AO Trauma CPP Bone Infection Registry (Morgenstern *et al*., 2021)] involving 292 patients, anti-Gmd antibodies were found to be absent in most patients with bone infection, and levels in serum at the time of initial infection correlated with a reduced chance of ADs at one-year follow-up (Kates *et al*., 2020). A follow-up analysis of specific antibody responses against 8 *S. aureus* antigens (Table 1) in the same patient cohort revealed that antibodies against IsdA and IsdB were associated with poor clinical outcomes including amputation, septic death, and elevated antibody levels against SCIN, CHIPS, Gmd, Amd, and Hla correlated with a reduced chance of ADs at one-year follow-up (Muthukrishnan *et al*., 2021). However, the presence of high levels of anti-*S. aureus* antibody in the serum of a small subset of these patients did not confer protection, which raises the question of the IgG subclass of these antibodies and their functionality in these patients.

Human IgG is comprised of four distinct subclasses IgG1, IgG2, IgG3, and IgG4 with well-defined effector functions (Jefferis and Kumararatne, 1990; Vidarsson *et al*., 2014). The first two subclasses make up 80 to 90 % of total IgG in human serum and are the most effective at combating infections. IgG4 normally represents only about 3 to 6 % of total IgG. However, higher concentrations of IgG4 have been observed in long-term and repeated exposure to *S. aureus* antigens (Aalberse *et al*., 1983; Sigal, 2012; Swierstra *et al*., 2015). Since IgG4 is less effective in mediating effector functions and does not mediate opsonisation or complement fixation, a shift away from production of the more protective subclasses towards IgG4 could be immunosuppressive during *S. aureus* infection. Class-switching to IgG4 could be yet another tool in the considerable arsenal of *S. aureus* (Muthukrishnan, *et al*., 2019).

To address these important questions of humoral immunity and immunosuppression, the relative contribution of IgG4 to total IgG anti-*S. aureus* levels were assessed in osteomyelitis patients at the time of their infection debridement surgery (fracture site debridement, joint replacement explant, bony debridement), and the relationship of this baseline humoral response with clinical outcome at 1 year. Specifically, the following hypotheses were tested: 1) anti-Gmd antibodies in osteomyelitis patients are disproportionately IgG4; and 2) the IgG4 to total IgG ratio of anti-*S. aureus* antibodies at the time of surgery is prognostic of clinical outcome, in which lower ratios are associated with IC, and higher ratios are associated with adverse events including septic death.

## Materials and Methods

### Human subjects

All human subject research was performed with informed consent under IRB-approved protocols (HM20009308, 20006017, and NCT01677000). Uninfected serum samples for this study were randomly chosen from healthy non-infected preoperative orthopaedic surgery patient cohort to serve as controls (*n* = 92, Ctrl). Serum samples (*n* = 135) obtained from patients with culture confirmed *S. aureus* bone infection at the time of their debridement surgery for infection treatment (fracture site debridement, joint replacement explant, bony debridement), and known 1-year clinical outcome of the surgery, were obtained from the AO Trauma CPP Bone Infection Registry. This cohort contained patients whose infection was documented as “cured” or “controlled” by the treating physician at 1-year post-op (IC, *n* = 95), and AD patients who had documentation of fracture non-union, infection persistence, septic death, amputation, or definitive surgery (joint arthrodesis, joint explanted) at 1-year (AD, *n* = 40). To assess antibody levels in the most severe infection condition, serum samples from patients who died from *S. aureus* sepsis and multiorgan failure were obtained immediately PM (< 3 h after death, PM, *n* = 7).

### Luminex-based immunoassays

IgG serum antibodies were measured using a custom Luminex^™^ assay following methodology previously described (Kates *et al*., 2020; Lee *et al*., 2020; Muthukrishnan *et al*., 2020; Nishitani *et al*., 2015; Oh *et al*., 2018). 8 immunodominant *S. aureus* antigens (Table 1) were investigated; 3 iron scavenging Isd proteins: (IsdA, IsdB, and IsdH), 3 virulence and immune evasion secretory proteins: CHIPS, Hla, and SCIN, and 2 cell wall Atl domains: Amd and Gmd. Monoclonal antibodies exist for Gmd and have allowed quantification of total IgG concentrations in this cohort (Lee *et al*., 2020) but monoclonal antibodies are not available for the other 7 antigens. Thus, total IgG and IgG4 levels are reported in this study as MFI units for each antigen at a constant serum dilution level to evaluate differences among groups. Total IgG levels (MFI) were also tested against tetanus toxoid as an irrelevant non-*S. aureus* control antigen to look for differences between the control and infection groups.

As in previous studies, each biotinylated antigen was coupled with distinct bead regions of avidin-coated magnetic beads (MagPlex-Avidin, Luminex Corp, Austin, TX, USA) at a density of 50 pmol/million beads. 50 μL containing 1,000 coupled beads were then mixed with 50 μL of 1:5,000 diluted sera per well yielding 100 μL of 1:10,000 diluted sera with 1,000 beads per well and were incubated at room temperature with shaking for 2 h. A wash step followed to remove unbound sera and then 100 μL of 1:500 diluted phycoerythrin-conjugated goat anti-human IgG reagent (Cat. #2040-09, Southern Biotech, Birmingham, AL) was added as the detection antibody and incubated at room temperature with shaking for 1 h. Afterward, the plate was washed and 130 μL of PBST-BSA [PBS (Cat. #P3813), with 0.1 % BSA (Cat. #A7888), and 0.1 % TWEEN^®^ 20 (Cat. #P9416), all from Sigma-Aldrich] was added and beads were resuspended by 2 min of shaking and mixing by pipette before measurement of total IgG bound to each antigen using a Luminex 200^™^ (xPONENT v3.1, Luminex Corp.). For IgG4 levels, the same process as just described was followed except serum was diluted at 1:500 before adding the bead mix (1:1,000 dilution after bead addition) and the detection antibody was replaced with 1:50 diluted phycoerythrin-conjugated mouse anti-human IgG4 Fc reagent (Cat. #9200-09, Southern Biotech).

### Statistical data analyses

**Fig. 1.**
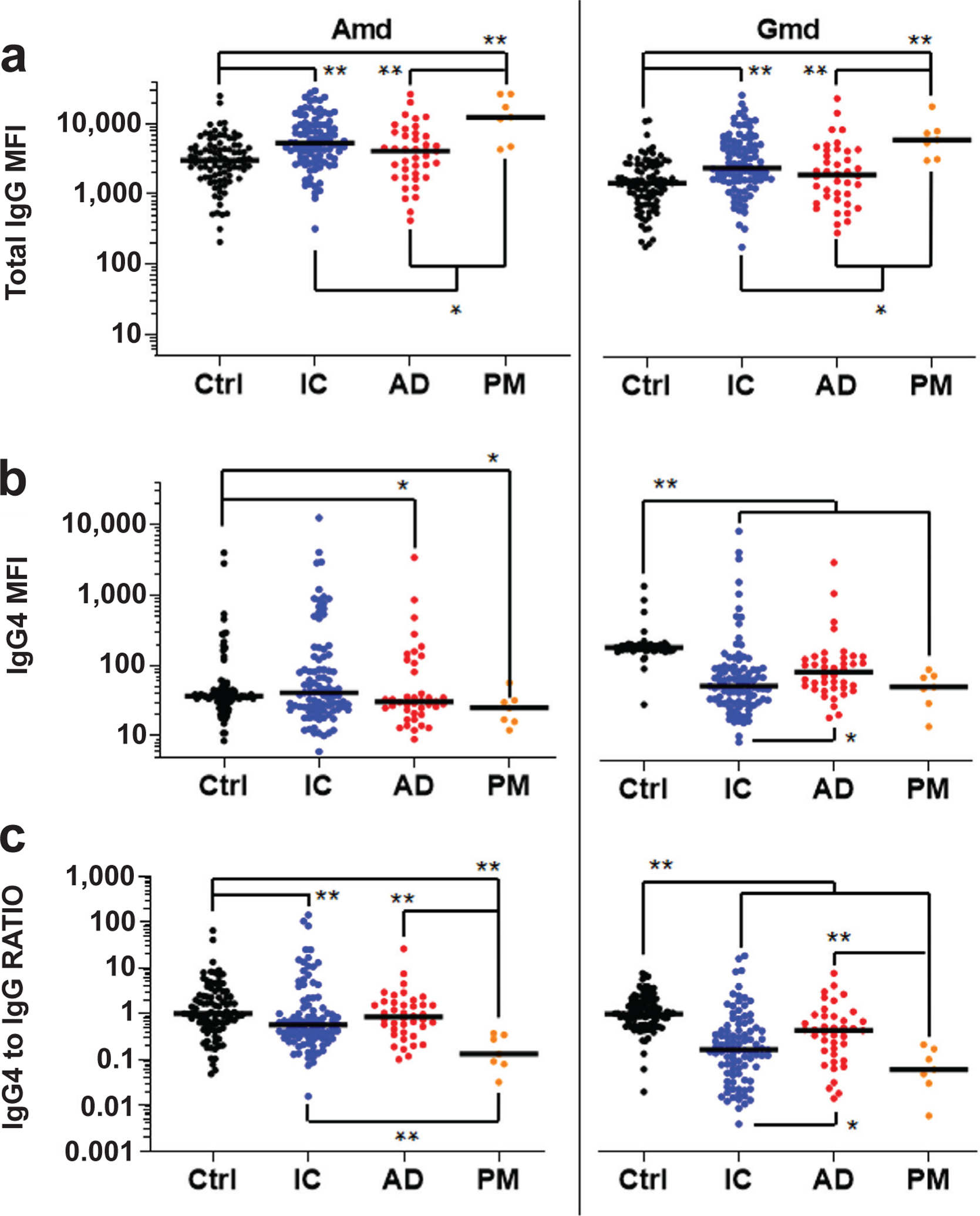
Anti-Atl IgG4 antibody levels are decreased in bone infection patients. AO Trauma CPP Bone Infection Registry baseline sera from uninfected patients (Ctrl, *n* = 92), infection controlled (IC, *n* = 95) patients, patients with adverse outcomes (AD, *n* = 40, fracture present, infection present, septic death, amputation, and definitive surgery at 1 year), and PM (*n* = 7) blood samples from osteomyelitis patients that died from *S. aureus* sepsis, were analysed for IgG antibodies specific for Amd and Gmd using a Luminex^™^ to determine: (**a**) total IgG MFI, (**b**) MFI for IgG4 specific antibodies, and (**c**) IgG4 MFI divided by total IgG MFI (RATIO). The data are presented for each patient with the median for the group. IgG4 to IgG RATIO represents the MFI for IgG4 divided by the MFI for total IgG normalised by Ctrl ratios such that the Ctrl median ratio is a value of 1. *p < 0.05, **p < 0.01, Wilcoxon rank-sum tests with adaptive Hochberg multiplicity adjustment.

**Fig. 2.**
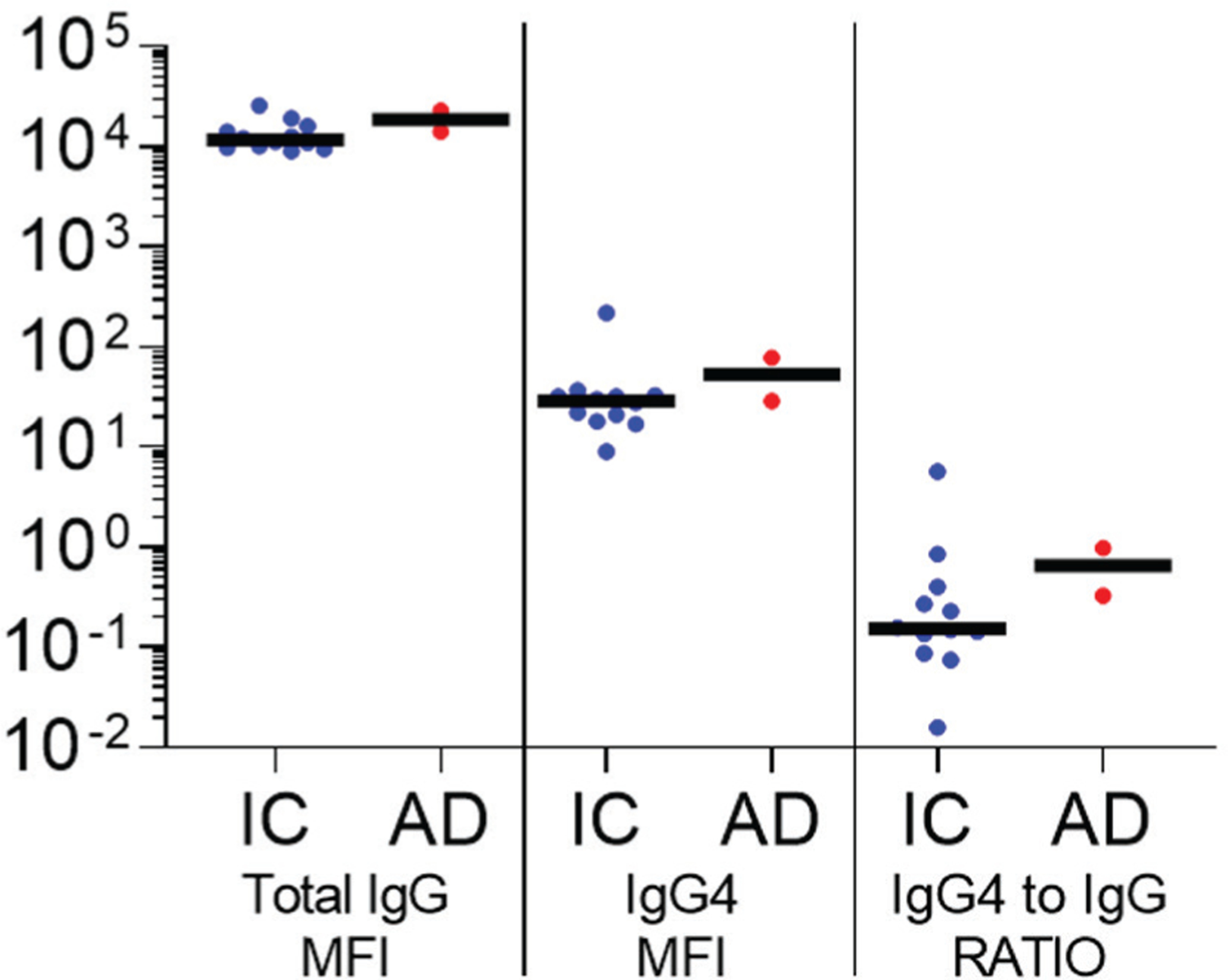
Initial screen of serum anti-Gmd IgG4 *vs*. total anti-Gmd IgG levels in *S. aureus* bone infection patients with high levels against Gmd. AO Trauma CPP Bone Infection Registry serum samples from patients with high anti-Gmd IgG levels (> 10 μg/mL) obtained at the time of their surgery for culture confirmed *S. aureus* bone infection (baseline) were divided into 2 groups: infection controlled at 1-year post-operation (IC, *n* = 12), and adverse outcome within 1 year (AD, *n* = 2, 1 knee fusion and 1 wound breakdown). The sera were analysed for IgG antibodies specific for Gmd using a Luminex^™^ to determine: (left) total IgG MFI, (centre) MFI for IgG4 specific antibodies, and (right) IgG4 MFI divided by total IgG MFI (RATIO). The data are presented for each patient with the median for the group without statistical analysis due to the small sample size.

**Fig. 3.**
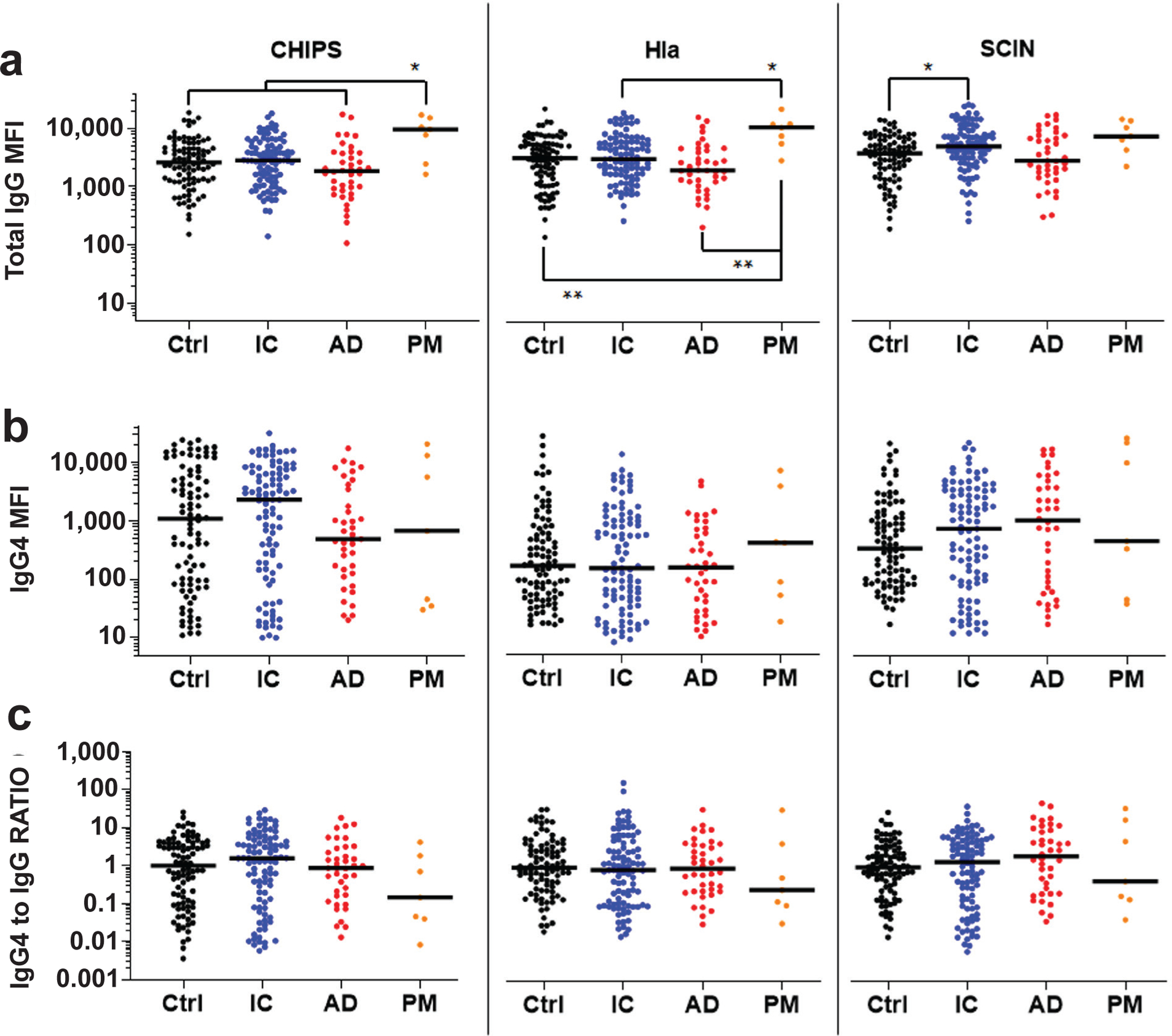
IgG4 antibodies against CHIPS, Hla and SCIN are not elevated in bone infection patients. The sera described in Fig. 1 were analysed for IgG antibodies specific for CHIPS, Hla and SCIN using a Luminex^™^ to determine: (**a**) total IgG MFI, (**b**) MFI for IgG4 specific antibodies, and (**c**) IgG4 MFI divided by total IgG MFI (RATIO). The data are presented for each patient with the median for the group. IgG4 to IgG RATIO represents the MFI for IgG4 divided by the MFI for total IgG normalised by Ctrl ratios such that the Ctrl median ratio is a value of 1. *p < 0.05, **p < 0.01, Wilcoxon rank-sum tests with adaptive Hochberg multiplicity adjustment.

**Fig. 4.**
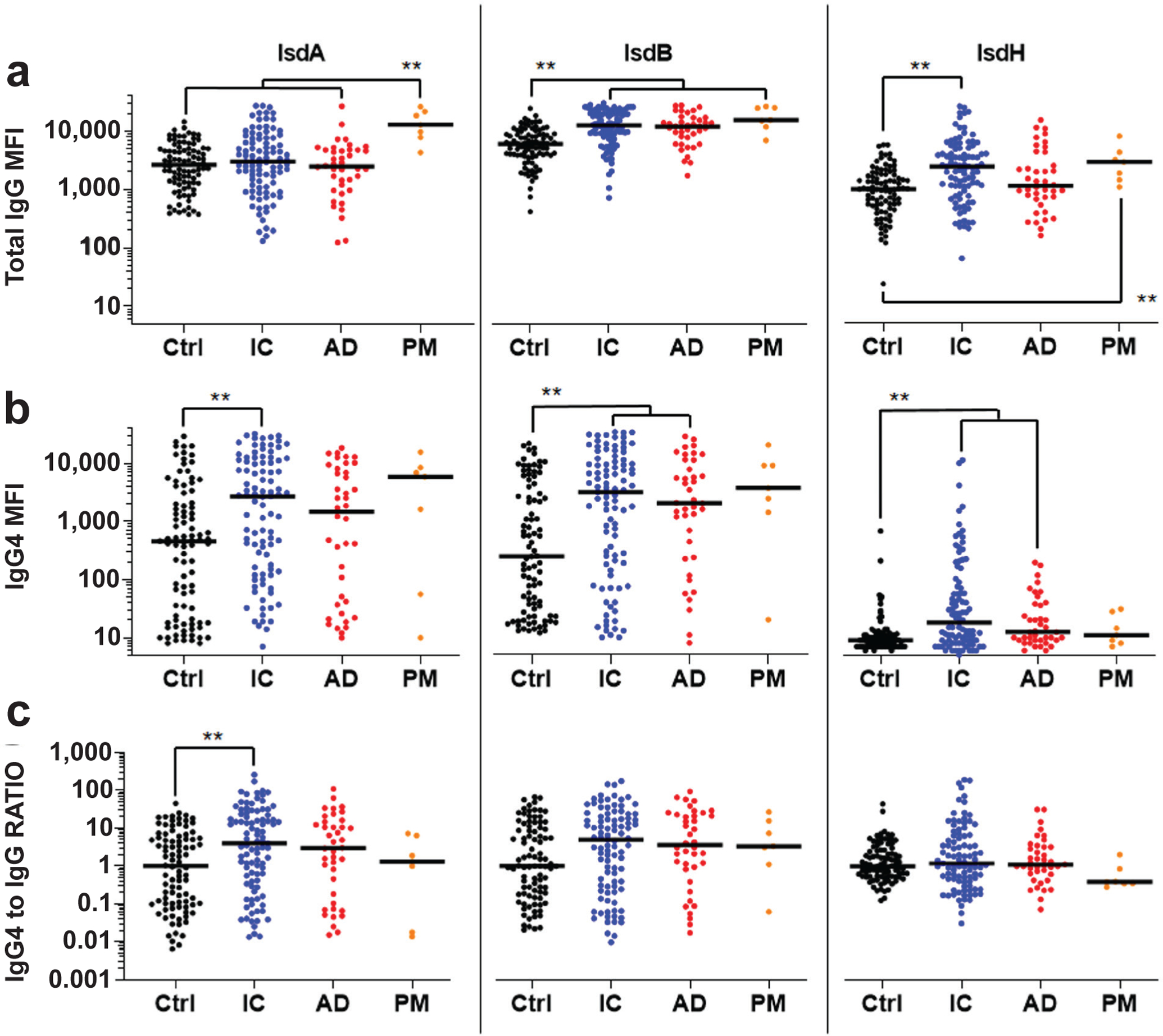
Elevated anti-Isd IgG4 antibody levels in bone infection patients. The sera described in Fig. 1 were analysed for IgG antibodies specific for IsdA, IsdB, and IsdH using a Luminex^™^ to determine: (**a**) total IgG MFI, (**b**) MFI for IgG4 specific antibodies, and (**c**) IgG4 MFI divided by total IgG MFI (RATIO). The data are presented for each patient with the median for the group. IgG4 to IgG RATIO represents the MFI for IgG4 divided by the MFI for total IgG normalised by Ctrl ratios such that the Ctrl median ratio is a value of 1. **p < 0.01, Wilcoxon rank-sum tests with adaptive Hochberg multiplicity adjustment.

Since monoclonal antibody concentrations could not be quantified for total IgG nor IgG4 for the *S. aureus* antigens of interest, the percent composition of IgG4 in total IgG could not be directly measured. Therefore, ratios of MFI values for IgG4 at the 1,000-fold dilution divided by MFI values for total IgG at a 10,000-fold dilution were used to assess relative contribution of IgG4. For each sample, the MFI value for IgG4 at 1:1,000 dilution was divided by the MFI value for total IgG at 1:10,000 dilution. Once all sample ratios were calculated, the ratios were then normalised to the control ratios such that normalised control ratios had a median value of 1 for each antigen (Fig. 1,2,3,4). The normalised ratios (IgG4 to IgG RATIO) for each antigen were then analysed using Wilcoxon rank-sum tests for pairwise comparison of groups (Ctrl, IC, AD, and PM) with an adaptive Hochberg multiplicity adjustment applied to control the risk of false discovery. The same statistics were also applied to the MFI values for IgG4 and total IgG. Additionally, nonparametric Jonckheere-Terpstra tests were used to assess total IgG, IgG4, and IgG4 to IgG RATIO for a trend of increase (Ctrl < IC < AD < PM) or decrease (Ctrl > IC > AD > PM) across groups for each antigen. Trends across groups were quantified using Kendall’s τ-b nonparametric measure of association. Statistical significance for all tests was set at *p* < 0.05.

## Results

To test if IgG4 class-switching could explain susceptibility to *S. aureus* bone infection in patients whose anti-Atl antibodies were predicted to be protective based on pre-clinical research, the relative concentration of IgG4 and total IgG antibodies against Amd and Gmd in the 4 groups of patients were determined (Fig. 1). Anti-Gmd IgG4 and IgG4 to IgG RATIO levels were higher in AD *vs*. IC (*p* < 0.05). However, contrary to the working hypothesis of IgG4 immunosuppression, the results showed that anti-Atl IgG4 levels were not increased in any of the infected patient groups over Ctrl, and that anti-Gmd IgG4 levels in IC, AD, and PM were significantly decreased *versus* Ctrl (*p* < 0.01).

To further assess the potential role of IgG4 immunosuppression in a sub-group of osteomyelitis patients with high levels of anti-Gmd antibodies in their serum, 14 samples containing > 10 μg/mL of anti-Gmd total IgG were assessed for IgG4 levels. Fig. 2 shows the results in which no differences were observed between the 12 IC and 2 AD patients studied. Although the limited sample size did not allow for powerful statistical analyses, the tight overlapping medians of total IgG, IgG4 and IgG4 to total IgG RATIO was found to indicate a remarkable lack of difference between groups.

IgG4 levels were also assessed against other antigens (Fig. 3), and the immunodominant Isd proteins associated with adverse events following *S. aureus* infection (Fig. 4). Consistent with the anti-Atl results, no increase of IgG4 antibodies against CHIPS, Hla and SCIN was found in any of the infected groups compared to Ctrl. There were also no differences in IgG4 to total IgG RATIO between any of these groups. In contrast, IgG4 antibodies against the Isd proteins were significantly elevated in IC *vs*. Ctrl for anti-IsdA, and in IC and AD *vs*. Ctrl for anti-IsdB and anti-IsdH IgG4 antibodies. Interestingly, there were no differences in IgG4 to total IgG RATIO between any of the Isd groups except for the significant increase in the anti-IsdA IC *versus* Ctrl.

Collectively, the aforementioned findings were consistent with the known relative immunogenicity of these *S. aureus* antigens, and a lack of disproportionate IgG4 class switching that could explain susceptibility to infection. However, to assess the potential role of IgG4 in osteomyelitis severity and clinical outcome following surgery, Jonckheere-Terpstra tests were performed as well as computed estimates of Kendall’s τ-b, which ranges from − 1 to 1 with a positive τ indicating a hypothetical increased severity of infection (Ctrl < IC < AD < PM), and a negative value occurring for a hypothetical decreased severity of infection (Ctrl > IC > AD > PM). The results (Table 2) show that the only significantly increased IgG4 levels across the infection spectrum were against Isd antigens, and the only significantly decreased levels were against Atl antigens.

**Fig. 5.**
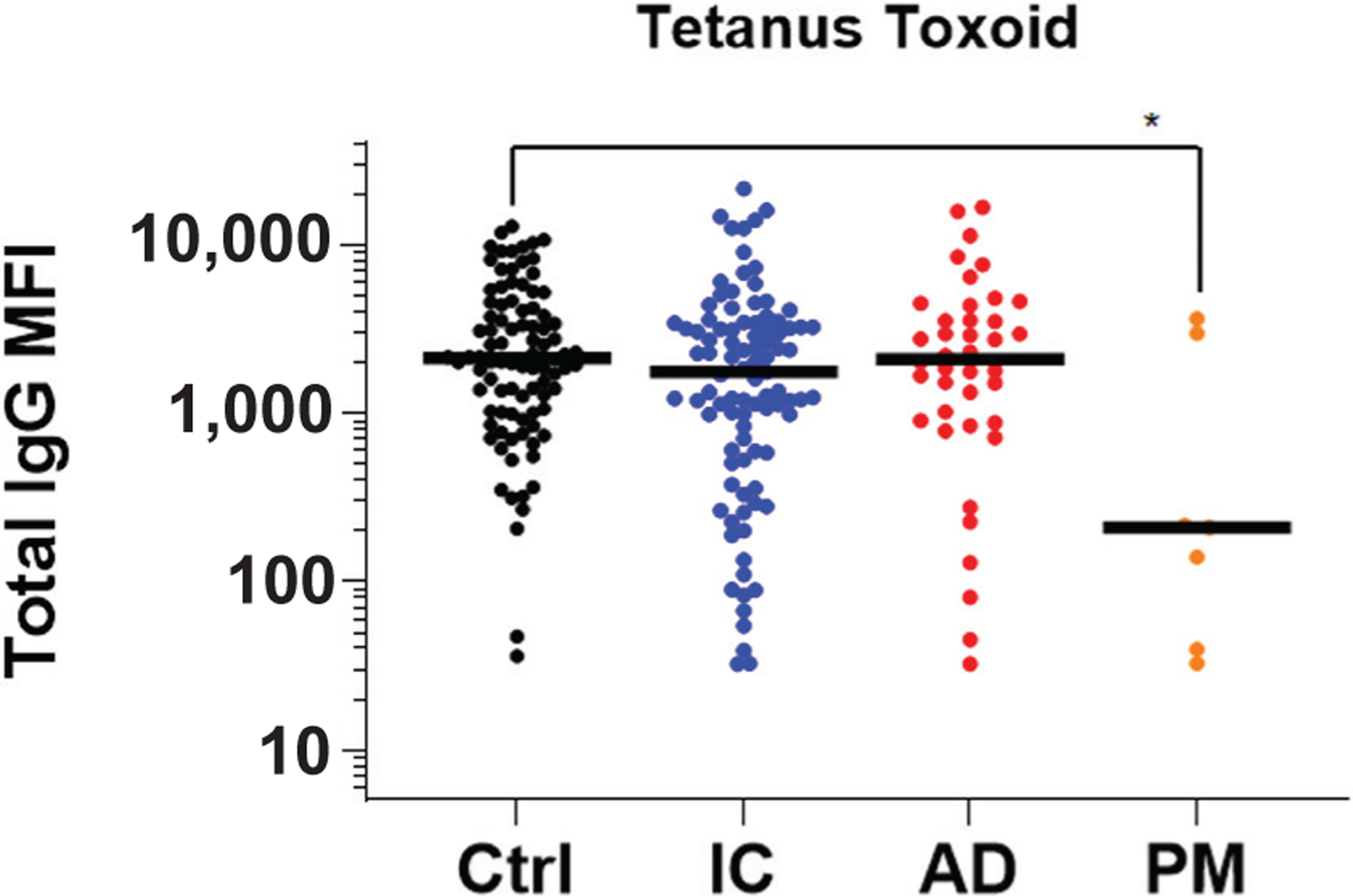
Anti-tetanus toxoid total IgG antibody levels are comparable across groups. The sera described in Fig. 1 were analysed for IgG antibodies specific for tetanus toxoid using a Luminex^™^ to determine total IgG MFI. Lower levels of antibodies against tetanus toxoid in the PM group is likely reflective of this small group having neglected immunisation booster shots and were not statistically different from the other infection groups. The data are presented for each patient with the median for the group. *p < 0.05, Wilcoxon rank-sum tests with adaptive Hochberg multiplicity adjustment.

Finally, total IgG levels were measured against tetanus toxoid as an irrelevant non-*S. aureus* control antigen. This only showed a difference between the control and PM infection group (*p* < 0.05) (Fig. 5). Lower levels of antibodies against tetanus toxoid in the PM group is likely reflective of this small group having neglected immunisation booster shots and were not statistically different from the other infection groups.

## Discussion

Rigorous clinical studies have established that the infection rate following elective orthopaedic surgery cannot be reduced below ~ 1 %, and that there are host specific factors that render this small population susceptible to infection (Masters *et al*., 2019; Ricciardi *et al*., 2020; Schwarz *et al*., 2019). While several comorbidities (*e.g*. obesity, diabetes, age) are known susceptibility factors (Schwarz *et al*., 2019), the assessment of the 292 osteomyelitis patients in the AO Trauma CPP Bone Infection Registry failed to identify differences in patient demographics (body mass index > 40 kg/m^2^, diabetes status, age, sex, Charlson Comorbidity Index of > 1, and Cierny-Mader host type) between infection and Ctrl groups, and these risk factors were not associated with adverse events (Kates *et al*., 2020). In contrast, several host immune responses were found that were associated with infection and clinical outcome. Specifically, there was a 51–69 % reduction in AD risk for every 10-fold increase in initial IgG concentration against Gmd, Amd, IsdH, CHIPS, SCIN, and Hla (*p* < 0.05) (Muthukrishnan *et al*., 2021). In contrast, anti-IsdB antibodies remained elevated in patients with ADs, and for every 10-fold change in the ratio of circulating anti-Isd to anti-Atl IgG at enrolment, there was a trending 2.6-fold increased risk (odds ratio = 2.555) of an adverse event (*p* = 0.105). Moreover, antibody increases over time correlated with ADs and decreases with positive outcomes. These studies demonstrate the potential of the humoral immune response against *S. aureus* as a prognostic indicator for assessing treatment success and identifying patients requiring additional interventions. Most notable was that only 6.7 % of the osteomyelitis patients had high levels of serum anti-Gmd antibodies (> 10 ng/mL) at the time of surgery, and that assessment of anti-Gmd antibody levels as a continuous variable showed a 60 % reduction in adverse-event odds (*p* = 0.04) for every 10-fold increase in concentration. Moreover, patients with low anti-Gmd levels demonstrated a significant 2.68-fold increased odds of ADs (*p* = 0.008). Given the prior findings that neutralising anti-Gmd antibodies aggregate *S. aureus* bacteria and facilitate opsonophagocytosis of these aggregates (so called megaclusters) *in vitro* (Varrone *et al*., 2014; Varrone *et al*., 2011), and passive immunisation with anti-Gmd antibodies protects mice from *S. aureus* osteomyelitis (Varrone *et al*., 2014; Yokogawa *et al*., 2018), it was reasoned that high levels of anti-Gmd antibodies in people are also protective, although the mechanism for protection from anti-Gmd antibodies is yet to be defined. However, infection and adverse events in patients with high levels against Gmd were not explained. Thus, this study aimed to test the hypothesis that patients who suffer ADs following re-implantation surgery are vulnerable to re-infection because their immune response is compromised by a shift in the antibody response from opsonising and complement-fixing IgG1 to IgG4 that does neither.

Others have examined potential roles for IgG4 in other categories of *S. aureus* infections including skin, bone, and lung infections as well as nasal polyps associated with idiopathic asthma. In each case prominent IgG4 responses to particular sets of secreted *S. aureus* antigens were observed. In idiopathic asthma, the Slps were identified as prominent inducers of a type 2 immune response featuring production of both IgE and IgG4 (Stentzel *et al*., 2017). In a broader examination among patients experiencing *S. aureus* infections of the skin, bones, or lungs, IgG4 responses to a variety of secreted products were observed particularly against the leukocidins and the SSLs (Swierstra *et al*., 2015).

In contrast to the primary hypothesis, it was found that anti-Gmd IgG4 levels and anti-Gmd IgG4 to total IgG RATIO in osteomyelitis patients were lower than Ctrl (Fig. 1), and that anti-Gmd IgG4 antibodies decreased in the hypothetic model of infection severity (Table 2). Of note is that this IgG4 to total IgG RATIO also held for the other 7 antibody responses against *S. aureus* antigens studied. Although this study had several limitations pertaining to patient data curation from several cohorts in distinct geographical locations, and imperfections in the duration course of infection (acute *vs*. chronic) and the case-matched controls, it was found that IgG4 class-switching was not a dominant mechanism of immunosuppression during *S. aureus* bone infection, and that further testing of this hypothesis in patients was not warranted.

There are 4 alternative explanations that might account for the lack of protection from *S. aureus* bone infection in patients with high anti-Gmd levels. The first is that they may have had low levels of anti-Gmd antibody at the time of infection, and subsequently developed high levels after a long chronic infection period prior to surgery. Given this open possibility, future clinical studies should be carefully designed to obtain information on the timing between suspected infection initiation and surgery. A second possibility is that other susceptible components of the immune proteome overwhelm anti-Gmd efficacy. As it is now known that some antibodies against *S. aureus* antigens exacerbate surgical-site infections by Trojan horse leukocyte formation and dissemination of the bacteria (Nishitani *et al*., 2020). Thus, preclinical studies are warranted to gain a greater understanding of the protective *vs*. pathogenic effects of functional antibodies to fully interpret the diagnostic and prognostic potential of circulating anti-Gmd antibodies. A third possibility is that the anti-Gmd antibodies in the bone-infection patients with high anti-Gmd levels are non-neutralising. Gmd is an enzyme that degrades the bacterial cell wall, and it has been shown that monoclonal antibodies against this protein can be either neutralising (inhibit enzymatic activity), or non-neutralising (bind to Gmd but do not inhibit enzymatic activity) (Gedbjerg *et al*., 2013). Given the clinical significance of this question as it pertains to active and passive immunisation against Gmd, this is currently being investigated with *in vitro* assays. Lastly, the original assumption that anti-Gmd antibodies are protecting by means of opsonisation may not be true, as previous studies of opsonising antibodies in *S. aureus* vaccine trials have not shown that opsonising antibodies are protective (Miller *et al*., 2020). Thus, it is possible that the anti-Gmd antibody response observed is a surrogate for a yet to be defined mechanism of protection.

## Conclusions

A human immune proteome against *S. aureus* exists in which the most immunodominant antigens appear to be against Isd proteins, and antibodies against these proteins are uniformly increased in osteomyelitis patients across all IgG classes including IgG4. In contrast, there is a lack of humoral immunity against Atl antigens across all IgG classes in osteomyelitis patients. Thus, infection and ADs in the small population of osteomyelitis patients with high levels against Gmd cannot be explained by IgG4 class-switching.

## Figures and Tables

**Table 1:** *S. aureus* antigens studied and their associated functions.

*S. aureus* antigens	Functions
**Iron scavenging**	Iron/haeme uptake and transport. Resistance to neutrophil killing.
IsdA	
IsdB	
IsdH	
**Virulence and immune evasion**	Inhibit complement activation. Prevent chemotaxis of neutrophils. Pore-forming toxin. Alter immune response.
SCIN	
CHIPS	
Hla	
**Cell wall enzymes**	Peptidoglycan hydrolases. Cell separation.
Amd	
Gmd	

**Table 2.** IgG4 antibodies against Isd proteins are increased while IgG4 antibodies against Atl proteins are decreased in patients with *S. aureus* bone infection. Nonparametric Jonckheere-Terpstra tests were performed on the serology described in Fig. 1,3,4, and Kendall’s τ-b was estimated for each antigen. τ ranges from − 1 to 1 with a positive τ indicating an increasing order (Ctrl < IC < AD < PM), and a negative value occurring for a decreasing order (Ctrl > IC > AD > PM). τ values in each cell are presented with Jonckheere-Terpstra test *p*-values in, where statistically significant *p*-values are in **bold.** Note that the only significantly increased IgG4 levels across the infection spectrum were against Isd antigens, and the only significantly decreased levels were against Atl antigens.

	IsdA	IsdB	IsdH	CHIPS	Hla	SCIN	Amd	Gmd
Total IgG	0.067	0.327	0.186	− 0.016	0.002	0.083	0.202	0.213
*p* values	0.18	**< 0.0001**	**0.0002**	0.75	0.98	0.10	**< 0.0001**	**< 0.0001**
IgG4	0.139	0.196	0.205	− 0.031	− 0.028	0.097	− 0.072	− 0.414
*p* values	**0.0058**	**0.0001**	**< 0.0001**	0.55	0.58	0.05	0.15	**< 0.0001**
IgG4 to IgG RATIO	0.113	0.114	− 0.009	− 0.025	− 0.044	0.068	− 0.148	− 0.341
*p* values	**0.0260**	**0.0244**	0.86	0.62	0.38	0.18	**0.0035**	**< 0.0001**

## Discussion with Reviewer

**Ito Hiromu**: This is an interesting study, but it appears to remain to be investigated what humoral immunity and immunosuppression in bone infection by *S. aureus* is all about, especially in relative contribution of antibodies against the specific antigens. Please describe possible examinations for future research.

**Authors**: Thanks for this excellent suggestion. The contribution of humoral immunity and immune suppression during *S. aureus* bone infection is a broad topic and one that is of great interest to us (Masters *et al*., 2019; Muthukrishnan *et al*., 2019). Our group and others are exploring these concepts mechanistically using mouse models of *S. aureus* osteomyelitis and infected patient samples. In particular, we are interested in assessing the frequencies of protective and pathogenic antibody-producing cells (circulating plasmablasts, memory B cells, long-lived plasma cells in the bone marrow) from *S. aureus* infected patients as a surrogate for susceptible *vs*. protective humoral immune proteome. Additionally, we are also exploring whether antigen-specific B cell anergy contributes to the differences in antibody responses. We hope to elaborate on these mechanisms in future publications. Here, for the sake of succinctness, we have discussed only relevant experiments pertaining to the anti-Gmd antibody responses.

## List of Abbreviations

AD: adverse outcome
Amd: amidase
Atl: autolysin
BSA: bovine serum albumin
CHIPS: *c*hemotaxis inhibitory protein from *S. aureus*
CPP: clinical priority program
Ctrl: healthy control
Gmd: glucosaminidase
Hla: *α*-haemolysin
IC: infection control
Isd: iron-surface determinant
MFI: median fluorescent intensity
PBS: phosphate buffered saline
PBST: phosphate buffered saline Tween
PM: post mortem
*S. aureus*: *Staphylococcus aureus*
SCIN: staphylococcal complement inhibitor
Slps: serine protease-like proteins
SSLs: staphylococcal superantigen-like proteins
